# Dendritic cell-derived MYD88 potentiates as a biomarker for immune regulation in hepatocellular carcinoma and may predict a better immunological result

**DOI:** 10.3389/fcell.2025.1554705

**Published:** 2025-03-24

**Authors:** Zheming Liu, Hengbo Zhu, Fengxia Zhang, Wenting Huang, Shipeng Zhu, Songjiang He, Yi Yao, Qibin Song, Xue Zhang

**Affiliations:** ^1^ Cancer Center, Renmin Hospital of Wuhan University, Wuhan, China; ^2^ Wuhan University Shenzhen Research Institute, Shenzhen, China; ^3^ Department of Pediatrics, Renmin Hospital of Wuhan University, Wuhan University, Wuhan, China; ^4^ Department of Rehabilitation Medicine, Renmin Hospital of Wuhan University, Wuhan, China; ^5^ Department of Pathology, Renmin Hospital of Wuhan University, Wuhan, China; ^6^ Department of Breast, Renmin Hospital of Wuhan University, Wuhan, China

**Keywords:** dendritic cells, MyD88, hepatocellular carcinoma, TME, immunotherapy

## Abstract

**Introduction:**

MYD88 (myeloid differentiation primary response 88) is a key adaptor protein mediate immune responses, primarily through Toll-like receptors (TLRs) and interleukin-1 receptor (IL-1R) signaling. The TLR/MYD88 pathway plays a critical role in dendritic cells (DC) maturation and function, contributing to the body’s innate immunity. Recent studies have further highlighted MYD88’s pivotal role in intrinsic immunity and its regulatory influence on the tumor microenvironment (TME) in hepatocellular carcinoma (HCC). The expression of MYD88 in DCs and its regulatory role in the TME have gained increasing attention.

**Methods:**

RNA-sequencing data retrieved from the TCGA and GEO databases were utilized for both the training and validation of our signature. Single-cell RNA transcriptome data from GEO were analyzed to investigate the correlation among subclusters of T cells, myeloid cells, and dendritic cells (DCs) within the HCC tumor microenvironment (TME). A combination of bioinformatics and machine learning approaches was employed to perform statistical analyses.Additionally, flow cytometry was conducted to quantify T cell subtypes and assess biomarker expression in DCs. A BALB/c-derived xenograft mouse model was established to evaluate the functional role of MyD88 in tumor progression and immunotherapy response. Furthermore, immunohistochemical (IHC) staining was performed to reassess the biological effects of MyD88 in HCC patients undergoing immune checkpoint inhibitor (ICI) therapy.

**Results:**

Our pan-cancer data analysis further highlights the significant impact of MYD88 on clinical outcomes in HCC. Analysis of TCGA and GEO databases confirms that MYD88 serves as a key signaling molecule in DCs, reinforcing its critical role in immune regulation. Our *in vitro* experiments demonstrates that MyD88 modulates T cell function through DCs. *In vivo*, H22 tumor cells exhibited accelerated growth in MyD88 knockout mice and a reduced response to anti-PD-1 treatment, whereas wild-type mice showed the opposite trend.

**Discussion:**

These findings underscore the critical role of MYD88 in DC function, suggesting its potential as a biomarker for immunoregulation in HCC. By shaping the TME, MYD88 not only regulates the immune response in HCC but also influences patient clinical outcomes. Both ex vivo and *in vivo* experiments further validate that MYD88 impacts DC functionality, contributing to variations in HCC progression

## 1 Introduction

MYD88 is a key adaptor protein in Toll-like receptors (TLR) signaling, palying a crucial role in immune responses (Sun et al., 2023; Saikh, 2021; Fitzpatrick et al., 2020). Comprising 296 amino acid residues, MYD88 features two specialized domains: the death domain and the TIR domain (Hardiman et al., 1996). Dimerization of the TIR domain is essential for MYD88-mediated signaling, highliting its pivotal role in TLR pathways (Kawai and Akira, 2010). Among the 14 TLR isoforms, all-except TLR3-signal at least partially through MYD88. By recognizing pathogen-associated molecular patterns (PAMPs) and damage-associated molecular patterns (DAMPs), MYD88 transmits signals downstream, activating IRAK and TRAF6 kinases (Kiripolsky et al., 2020). This leads to the stimulation of the NF-kB and MAPK signaling pathways, which drive critical cellular effects (Fan et al., 2022; Weng et al., 2024). Notably, activation of these pathways has been linked to tumor progression and metastasis (Fan et al., 2013).

TLRs are the primary class of pattern-recognition receptors (PRRs) expressed on the surfaces of DCs. These receptors detect DAMPs, triggering the transcription and synthesis of inflammatory factors that promote DC maturation. This maturation process leads to the upregulation of co-stimulatory molecules, such as CD80 and CD86, which enhance the activation and proliferation of naïve T cells. As a result, adaptive immunity is initiated, playing a pivotal role in shaping tumor outcomes (Wang et al., 2024).

Recent findings suggest that MYD88, beyond its essential role in intrinsic immunity, is a critical factor in regulating of the TME (Liu et al., 2023; Huo et al., 2024). Studies have shown that blocking MYD88 signaling downstream of TLR4 disrupts DC function, skews T cell differentiation toward Th2 cell phenotypes, and promotes tumorigenesis (Ochi et al., 2012a). Additionally, MyD88-deficient (MyD88^−/−^) mice, when subjected to repeated azoxymethane (AOM) administration, exhibited a higher incidence of colitis-associated cancer (CAC) compared to wild-type mice (Rakoff-Nahoum and Medzhitov, 2007). HCC, the fourth leading cause of cancer-related deaths worldwide, accounts for approximately 800,000 deaths annually. In studies of human liver cancer, MyD88 has been shown to be highly expressed in liver cancer tissues compared to normal liver tissues (Liang et al., 2013). Furthermore, elevated MyD88 expression promotes tumor proliferation and metastasis by activating the PI3k/Akt signaling pathway and enhancing epithelial-mesenchymal transition (EMT) process (Jia et al., 2014). Research suggests that MARCHF3-mediated degradation of PARP1 in tumor cells activates the cGAS-STING pathway in DCs, thereby modulating antitumor immunity in HCC. Furthermore, *in situ* DC vaccination for HCC demonstrates substantial potential in modulating the TME (Lurje et al., 2021). In light of these findings, the expression of MYD88 in DCs and its role in TME regulation are emerging as critical areas of research, with significant implications for clinical outcomes in HCC.

We analyzed data from The Cancer Genome Atlas (TCGA) and Gene Expression Omnibus (GEO) to demonstrate that MYD88, a key signaling molecule in DCs, plays a critical role in DC function, highlighting its potential as a biomarker for immune regulation in HCC. By modulating the TME, MYD88 not only regulates the immune response in HCC but may also influence patient clinical outcomes. Both *ex vivo* and *in vivo* experiments have confirmed that MYD88 can produce variable effects in HCC by altering DC function. Moving forward, further investigation into the mechanisms by which MYD88 operates in DCs and the TME, as well as its potential applications in immunotherapy, may provide valuable insights and novel therapeutic targets for the treatment of HCC.

## 2 Materials and methods

### 2.1 Dataset preparation and preprocessing

#### 2.1.1 Acquisition of pan-cancer data

We obtained RNA sequencing (transcripts per million, TPM) gene expression data along with corresponding clinical information for TCGA pan-cancer and GTEx samples from UCSC Xena (https://gdc.xenahubs.net). The downloaded gene expression files contained pre-mapped gene symbols, which were further processed by converting ENSEMBL IDs to their corresponding gene symbols. In cases where multiple ENSEMBL IDs mapped to the same gene symbol, the expression value with the highest level was retained for subsequent analyses. To ensure the reliability of survival analyses, samples with incomplete survival information or a total survival time of zero were excluded.

#### 2.1.2 Acquisition of GEO data

We downloaded gene expression matrix files for GSE14520, GSE76297, and GSE76427 from the GEO database (http://www.ncbi.nlm.nih.gov/geo/). Subsequently, probe identifiers were converted to gene symbols. In cases where a probe mapped to multiple genes, the probe was removed, and the highest expression value among the correponding gene symbols was retained.

### 2.2 Univariate analysis and grouping

Univariate Cox analysis (P < 0.05) was performed to identify prognosis-related genes within the selected cohort. Patients were then stratified into high- and low-expression groups based on the gene expression levels. Overall survival (OS) between these groups was compared using the survminer package. Additionally, time-dependent receiver operating characteristic (ROC) curve analysis, conducted with the timeROC package, was used to evaluate the predictive performance of the identified prognostic features.

### 2.3 ssGSEA analysis

Single-sample gene set enrichment analysis (ssGSEA) is a widely utilized method for evaluating immune cell infiltration. This approach estimates the relative enrichment of a specific gene set, such as an immune cell gene set, within each sample by comparing its gene expression data to the overall transcriptomic expression profile of the sample. In the context of immune cell infiltration analysis, ssGSEA quantifies the relative abundance of various immune cell types in individual sample. The ssGSEA method converts the gene expression profile of each sample into gene set enrichment scores based on the input expression matrix and immune cell marker gene sets. These enrichment scores are used to assess the degree of immune cell infiltration. The immune gene sets employed in this study are derived from the research conducted by Charoentong et al. (2017).

The ssGSEA process begins by ranking all genes in descending order based on their expression levels. The cumulative distribution function is then computed for genes with higher expression within a predefined gene set, generating a metric known as the gene set enrichment score (GSE). For each sample, gene expression is ranked similarly, and a corresponding GSE is calculated at each position in the ranking. Finally, these scores are either averaged or weighted to drive a single ssGSEA score, representing the relative abundance of the immune cell type associated with the gene set. The ssGSEA analysis was conducted using the ssgsea function from the R package.

### 2.4 Single-cell analysis

The single-cell RNA sequencing (scRNA-seq) dataset was preprocessed using the Seurat R package (version 4.4.0). Data filtering was performed based on two parameters: min.cells = 3 and min.features = 200. Quality control was applied to the integrated dataset, ensuring that the proportion of mitochondrial genes did not exceed 5%. Counts were normalized using the “LogNormalize” method and scaled with the ScaleData function. Batch effects were corrected using Harmony. Cell Proximity was assessed with the FindNeighbors function, followed by clustering via the FindClusters function with a resolution of 0.6. The results were visualized using Uniform Manifold Approximation and Projection (UMAP). Differentially expressed genes across cell subpopulations were identified using the FindMarkers function.

### 2.5 Enrichment analysis

Gene Ontology (GO) analysis is a fundamental bioinformatics tool for annotating genes and their products across three primary categories: cellular components (CC), molecular functions (MF), and biological pathways (BP). The Kyoto Encyclopedia of Genes and Genomes (KEGG) is a comprehensive databases that provides insights into genomes, biological pathways, diseases, and chemicals. In this study, GO functional enrichment analysis and KEGG pathway analysis were performed using the clusterProfiler package to predict potential molecular functions of the genes of interest. A p-value of less than 0.05 was considered statistically significant.

### 2.6 Cell culture

The mouse hepatocellular carcinoma cell line H22 was obtained from the Shanghai Cell Bank at the Chinese Academy of Sciences. The cells were cultured in a CO_2_ incubator using RPMI-1640 complete medium. Once the cells reached 70%–80% confluence in the culture dish, they were passaged with a complete medium exchange. Cells in the logarithmic growth phase were used for the experiments.

### 2.7 Establishment of a mouse model of H22 cell-homocyte tumor

Male BALB/c mice and BALB/c (H-2d) Myd88^−/−^ mice, aged 6–8 weeks, were selected for the study. The right side of each mouse’s back was shaved and sterilized with alcohol, followed by subcutaneous inoculation of 100 μL (3 × 10^5^ cells) of H22 cells into the lower right back. Tumor formation was monitored daily, with tumor sizes typically reaching that of a grain of rice by day 5. Once tumors had developed, the inoculated mice were randomly assigned to intervention groups. Mice received either a MyD88 inhibitor (50 mg/kg) via intraperitoneal injection once daily, or a PD-L1 antibody (200 μg) via intraperitoneal injection every 3 days, while the control group received an equivalent volume of solvent intraperitoneally. The total observation period lasted for 3 weeks. At the end of the study, mice were deeply anesthetized with 1% pentobarbital sodium and euthanized under deep anesthesia. Spleens and local draining lymph nodes were harvested, and data on tumor size and body weight changes were recorded for statistical analysis.

### 2.8 Detection of treg and T cells by flow cytometry

The spleen was processed into a single-cell suspension using RPMI-1640 complete medium. Cells were stained with PE-labeled CD69 antibody and PE-Cy5-labeled CD3 antibody, followed by flow cytometric analysis using FlowJo software. Additionally, a separate spleen-derived single-cell suspension was prepared and labeled with FITC-labeled CD4 and APC-labeled CD25 antibodies for flow cytometric detection and analysis. Similarly, local draining lymph nodes were processed into single-cell suspensions, stained with a PE-labeled CD69 antibody and a PE-Cy5-labeled CD3 antibody, and analyzed by flow cytometry using FlowJo software.

### 2.9 Cultures and interventions for bone marrow-derived DCs

Bone marrow cells were harvested from the lower limb femur and tibia of BALB/c mice were taken and induced in culture using GM-CSF (final concentration 20 ng/mL) and IL-4 (final concentration 10 ng/mL) for induction.The cells were divided into four groups: a control group (without any treatment), an LPS intervention alone group, an LPS+10 μM MyD88 inhibitor group, and an LPS+40 μM MyD88 inhibitor group. Analyses were performed 48 h after the intervention using flow cytometry.

### 2.10 Lymphocyte mixed culture

Peripheral lymph nodes were harvested from C57BL/6 (B6, H-2b) mice. Lymphocytes were processed into single cell suspensions and labelled with CFSE. DCs, treated using the same method as described above, were co-cultured with these lymphocytes for 3 days. The cells were then labelled for CD4 and CD8 and analyzed using flow analyzed.

### 2.11 Detection of T-cell proliferation by BrdU assay

Lymph nodes from BALB/c mice were collected and processed into single-cell suspensions. The cells were plated into 96-well plates at a density of 2 × 10^5^ cells per well, with 100 μL of culture medium added to each well. Functional antibodies, anti-CD3e (final concentration: 2 mg/mL) and anti-CD8e (final concentration: 1 mg/mL), were added to each well. Additionally, MyD88 inhibitor was introduced at two different concentrations (10 μM and 40 μM), with each condition set up in duplicate. After 24 h of incubation, the 96-well plate was retrieved, and cell proliferation was assessed using the BrdU assay.

### 2.12 Statistical analysis

Statistical analyses were performed using R version 4.1.2. The Wilcoxon test was applied to compare statistical differences between two groups, while the Kruskal-Wallis test was used to evaluate differences among multiple groups. Survival curves were generated using the Kaplan-Meier method, and the log-rank test was conducted to compare overall survival differences between groups.

Univariate and multivariate analyses were performed using Cox regression models to evaluate the independent prognostic significance of risk scores in relation to other clinical characteristics. Receiver operating characteristic (ROC) curves were generated to assess the predictive performance of the risk model for 1-, 2-, and 3-year OS. Spearman correlation analysis was conducted to examine relationships between variables. A p-value of less than 0.05 was considered statistically significant. The following notations were used: **** for p < 0.0001, *** for p < 0.001, ** for p < 0.01, * for p < 0.05, and ns for non-significant results.

## 3 Results

### 3.1 MYD88 expression is dysregulated in pan-cancer and correlates with pathological clinical features of hepatocellular carcinoma

RNA sequencing (TPM) gene expression data for TCGA pan-cancer and GTEx samples, along with corresponding clinical information, were obtained from UCSC Xena (https://gdc.xenahubs.net). Additionally, bulk RNA-seq data for tumors and adjacent non-cancerous tissues from the GSE14520 and GSE76297 HCC datasets of hepatocellular carcinoma were retrieved from the GEO database. Differential expression analysis indicated that MYD88 expression varied significantly between normal and tumor samples across multiple cancer types, including ACC, BLCA, BRCA, and LIHC. Notably, MYD88 expression was found to be downregulated in tumor samples within the TCGA-LIHC dataset. In addition, comparison of MYD88 expression between normal and tumor samples from the GSE14520 and GSE76297 hepatocellular carcinoma datasets confirmed the downregulation of MYD88 in tumor samples, consistent with our findings in the TCGA-LIHC dataset (see Supplementary Figure S1A, B). Immunohistochemistry plots of MYD88 expression in normal and tumor samples of hepatocellular carcinoma were also retrieved from the Human Protein Atlas (HPA) database (see Supplementary Figure S1C). Statistical analyses of MYD88 expression based on clinical variables, including age, gender, stage, and grade, were performed in the TCGA-LIHC dataset; however, no significant differences were observed (see Supplementary Figure S1D).

**FIGURE 1:**
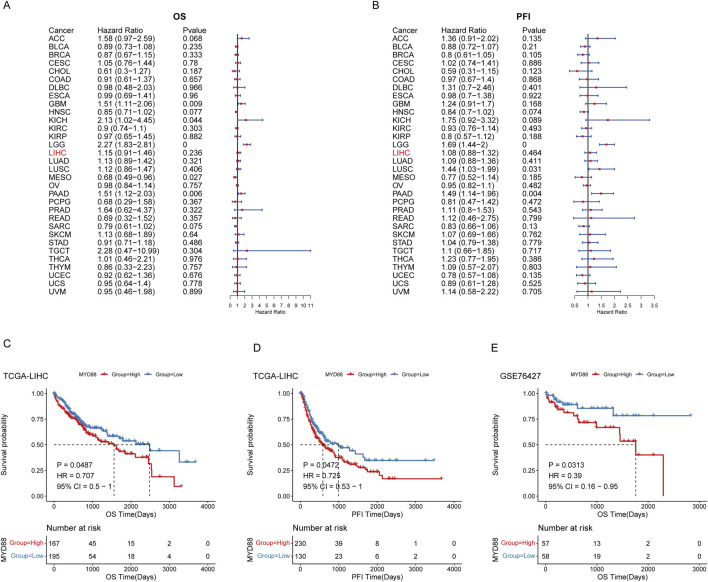
Prognostic analysis of MYD88. **(A)** Forest plot of OS in TCGA pan-cancer; **(B)** Forest plot of progression-free interval (PFI) in TCGA pan-cancer; **(C)** Kaplan-Meier curve for OS in TCGA-LIHC; **(D)** Kaplan-Meier curve for progression-free interval (PFI) in TCGA-LIHC; **(E)** Kaplan-Meier curve for OS in GSE76427.

Utilizing follow-up data from various cancer types in TCGA, we evaluated the impact of MYD88 expression on cancer prognosis and presented a univariate prognostic analysis forest plot for Overall Survival (OS) and Progression-Free Interval (PFI) (Figures 1A, B). TCGA-LIHC samples were categorized into high- and low-expression groups based on MYD88 expression, using the optimal cutoff determined by the ‘surv_cutpoint’ algorithm in the survminer R package. The Kaplan-Meier (KM) survival curves for OS and PFI were then plotted over time. The KM survival curves for OS and PFI exhibited statistically significant differences between the high- and low-expression groups (p < 0.05), indicating that patients with high MYD88 expression had significantly poorer OS and PFI compared to those with low expression (Figures 1C, D). Furthermore, MYD88 expression levels in GSE76427 were used to stratify samples into high- and low-expression groups. Prognostic analysis using the KM method demonstrated that patients with high MYD88 expression had significantly lower OS than those with low expression (p < 0.05) (Figure 1E).

### 3.2 Prognostic value of MYD88 in pan-cancer and may influence clinical outcome in hepatocellular carcinoma

The tumor microenvironment in HCC is highly complex, with patients often exhibiting impaired immune function within the TME. As demonstrated in the preceding analyses, samples with high MYD88 expression are associated with poorer OS. Thus, we investigated whether MYD88 serves as a reliable predictor of immunotherapy outcomes. To address this, we conducted an analysis using the HCC immunotherapy cohort GSE140901. Consistent with findings from the general cohort, samples with high MYD88 expression exhibited shorter survival (Supplementary Figure S2A). Additionally, MYD88 predicted immunotherapy response with an area under the curve (AUC) of 0.56 (Supplementary Figure S2B). Furthermore, we analyzed the correlation between MYD88 and immune checkpoints in the TGGA cohort. The results indicated a significant positive correlation between MYD88 and most immune checkpoints, suggesting that MYD88 may serve as a potential biomarker for predicting the immunotherapy responsiveness (Supplementary Figure S2C). To further investigate the relationship between MYD88 expression and clinical prognosis, we analyzed MYD88 levels in patients undergoing immunoneoadjuvant therapy. Our findings revealed that MYD88 expression was significantly higher in the immune response group compared to the low immune response group (34.49 ± 4.772 vs. 19.03 ± 2.767, p = 0.0216; Supplementary Material S1). These results suggest that MYD88 may enhance the efficacy of tumor immunotherapy, particularly PD-1/PD-L1 inhibitors, within the tumor microenvironment (Supplementary Material S1).

### 3.3 MYD88 correlates with TME cells

We anlayzed the infiltration of 29 immune cell types using ssGSEA based on TCGA-LIHC data. Differences in infiltration levels between high and low MYD88 expression subgroups were assessed using the rank sum test. Significant differences were observed in the infiltration of CD56 dim natural killer cells, central memory CD8+T cells, gamma delta T cells, immature dendritic cells, memory B cells, neutrophils, T follicular helper cells, and type 2 T helper cells between high and low MYD88 expression groups (see Supplementary Figure S3A). However, no significant differences were detected between the high- and low-expression subgroups when calculating immunity scores, stroma scores, tumor purity, and ESTIMATE scores using the ESTIMATE algorithm.

We performed Spearman correlation analyses to evaluate the relationship between MYD88 expression and ssGSEA immune cell scores, as well as immune scores, stromal scores, tumor purity, and ESTIMATE scores. Twelve immune cell scores exhibited significant correlations with MYD88 expression (p < 0.05). Specifically, the immune cell scores of activated CD8 T cell, CD56 bright natural killer cell, CD56 dim natural killer cell, and neutrophil showed negative correlations with MYD88 expression. In contrast, the scores for central memory CD4 T cells, central memory CD8 T cells, immature B cells, immature dendritic cells, memory B cells, and other immune cell types demonstrated positive correlations with MYD88 expression (see Supplementary Figures S3B, C).

**FIGURE 2:**
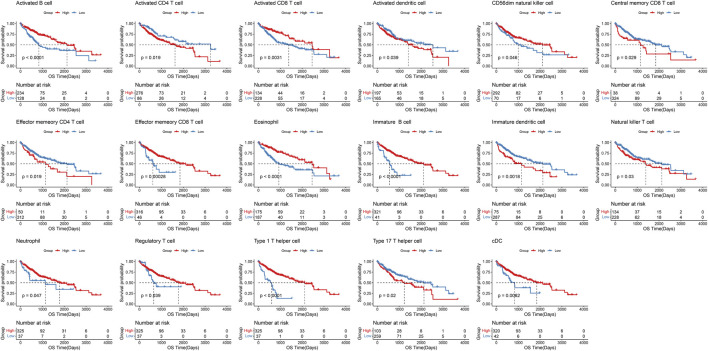
Kaplan-Meier curve for immune infiltration analyzed by ssGSEA.

Simultaneously, we employed the immune cell infiltration scores obtained from single-sample Gene Set Enrichment Analysis (ssGSEA) to assess the impact of immune cells on the overall survival (OS) prognosis of liver cancer (Figure 2). Patients were stratified into high and low immune infiltration groups based on the optimal threshold. Kaplan-Meier survival curves were generated to compare survival outcomes between these groups. The results indicated that the infiltration scores of 17 immune cell types were significantly associated with patient prognosis, including Activated B cells (p < 0.0001) and conventional dendritic cells (cDCs) (p = 0.0062).

### 3.4 Single-cell data analysis of hepatocellular carcinoma

To further investigate the role of MYD88 in the HCC microenvironment crosstalk, we selected hepatocellular carcinoma samples (excluding fetal liver samples) from the Hepatocellular Carcinoma Single Cell Sequencing dataset GSE156625. This dataset included tumor and paratumor samples from 14 HCC patients, along with one healthy liver sample (HN1), which contained a low cell count. The selection of GSE156625 from the datasets GSE149614, GSE156625, and GSE182159 was based on its highest number of cDCs in the tumor samples following cDC cell annotation.

The scRNA-seq dataset was preprocessed using the R package Seurat (version 4.4.0), with filtering applied based on two parameters: min.cells = 3 and min.features = 200 (see Supplementary Figures S4A, B). Following quality control, a total of 73,589 cells were retained, of which 57,254 were derived from tumor samples. These cells were classified into nine major cell types: T/NK cells, B cells, plasma cells, cycling cells, hepatocytes, endothelial cells, myeloid cells, fibroblasts, and mast cells (see Supplementary Figures S4C–E). In the HCC single-cell dataset, T/NK cells were the most predominant, followed by endothelial cells. Notably, the proportion of hepatocytes was significantly higher in tumor samples, but markedly lower in normal samples (see Supplementary Figure S4F).

**FIGURE 3:**
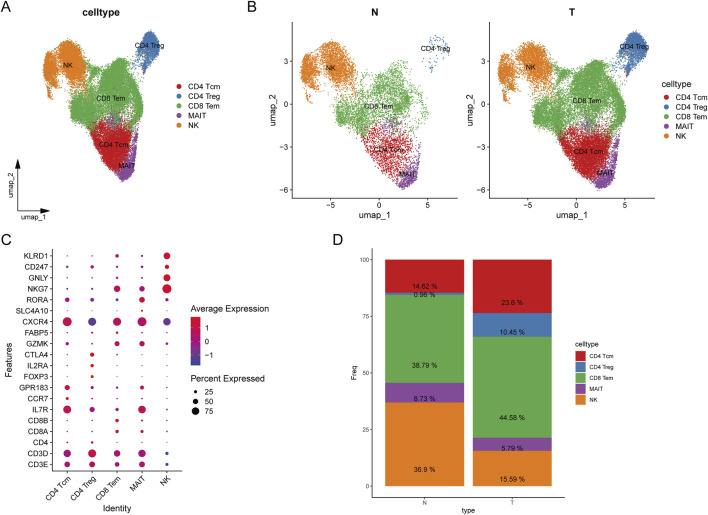
Classification of T cells. **(A)** UMAP plot of T cell clustering results; **(B)** UMAP plot of T cell clustering in tumors versus normal tissues; **(C)** Bubble plot of annotated markers for T cells; **(D)** Stacked bar plot of T cell proportions.

To further investigate the heterogeneity of T cell subpopulations and identify potential factors influencing their recruitment in HCC patients, we extracted T/NK cells and performed further fractionation for annotation. T cells were classified into CD8^+^ T cells based on the expression of CD8A and CD8B, into CD4^+^ T cells based on CD4 expression, and NK cells based on the expression of NKG7, GNLY, and KLRD1. Differential gene expression among the various clusters was analyzed using the FindAllMarkers function from the Seurat package, with the marker genes presented in Figure 3A. Ultimately, T/NK cells were subdivided into CD4^+^ central memory T cells (Tcm), CD4^+^ regulatory T cells (Treg), CD8^+^ effector memory T cells (Tem), mucosal-associated invariant T (MAIT) cells, and NK cells (Figure 3A).

In HCC tumor samples, the proportions of CD8^+^ Tem, CD4^+^ Tcm, and CD4^+^ Treg cells were significantly higher compared to normal samples, whereas the proportion of NK cells was higher in normal samples (Figure 3B). CD8^+^ Tem cells are characterized by long-term antigenic memory and rapid cytotoxic activity against tumor cells. Their increased presence in tumors suggests an active immune response attempting to counteract tumor progression, indicating potential long-term antigenic stimulation within the tumor microenvironment (TME). However, this could also reflect a mechanism of tumor immune evasion, wherein tumor cells suppress CD8^+^ T cell anti-tumor activity through various inhibitory signals. This bubble chart illustrates the distribution of differentially expressed genes across various cell types. The X-axis represents different T cell subtypes, while the Y-axis denotes differentially expressed genes. Our analysis revealed that CXCR4 is highly expressed across all T cell subtypes. Additionally, IL7R is predominantly expressed in CD4 central memory T cells (Tcm) and mucosal-associated invariant T (MAIT) cells, whereas NK cells exhibit high expression of NKG7, GNLY, and KLRD1 (Figure 3C). The increased proportions of CD8^+^ Tem, CD4^+^ Tcm, and CD4^+^ Treg cells in tumor samples indicate a complex immune response occurring within the TME, characterized by prolonged antigenic stimulation and significant immunosuppression. In contrast, the higher proportion of NK cells in normal samples suggests a more robust innate immune defense in a healthy state (Figure 3D). The reduction in NK cell numbers in tumor samples may be attributed to tumor immune evasion mechanisms. Overall, these findings highlight an immune imbalance within the tumor microenvironment, where effector immune responses are present but are suppressed by regulatory T cells and other immunosuppressive mechanisms, ultimately hindering complete tumor eradication.

**FIGURE 4:**
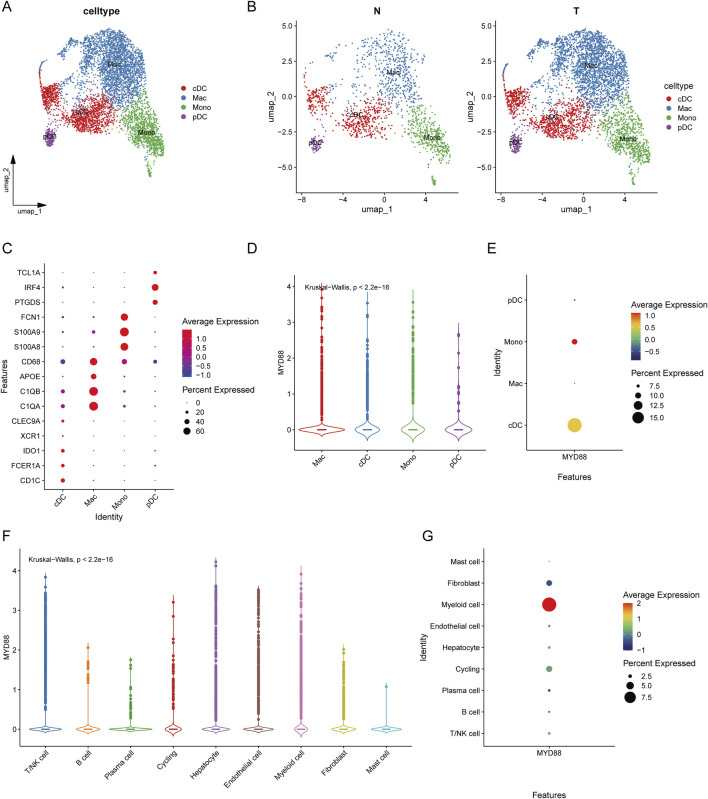
Classification of myeloid cells and expression of MYD88. **(A)** UMAP plot of myeloid cell clustering results; **(B)** UMAP plot of myeloid cell clustering in tumors versus normal tissues; **(C)** Bubble plot of annotated markers for myeloid cells; **(D)** Violin plot of MYD88 expression in myeloid cells; **(E)** Bubble plot of MYD88 expression in myeloid cells; **(F)** Violin plot of MYD88 expression in single cells; **(G)** Bubble plot of MYD88 expression in single cells.

Additionally, we further subdivided myeloid cells into cDCs, macrophages (Mac), monocytes (Mono), and plasmacytoid dendritic cells (pDCs), with respective markers indicated (Figure 4A). Figure 4B presents UMAP visualizations of tumor-associated and normal myeloid cell clusters. Figure 4C illustrates the cell type-specific expression patterns of various genes. CD68, APOE, C1QA, and C1QB are predominantly expressed in macrophages, while FCN1, S100A8, and S100A9 are specifically expressed in monocytes. Additionally, TCL1A, IRF4, and PTGDS show distinct expression in plasmacytoid dendritic cells (pDCs). Further subdivision of myeloid cells revealed that MYD88 was specifically expressed in monocytes and cDCs (Figures 4D, E), both of which play a crucial role in anti-tumor immunity. On the contratry, pDCs do not exhibit a significant anti-tumor effect. MYD88 was predominantly expressed in myeloid cells (Figures 4F, G).

### 3.5 Dendritic cell heterogeneity in the single-cell tumor microenvironment of hepatocellular carcinoma

**FIGURE 5:**
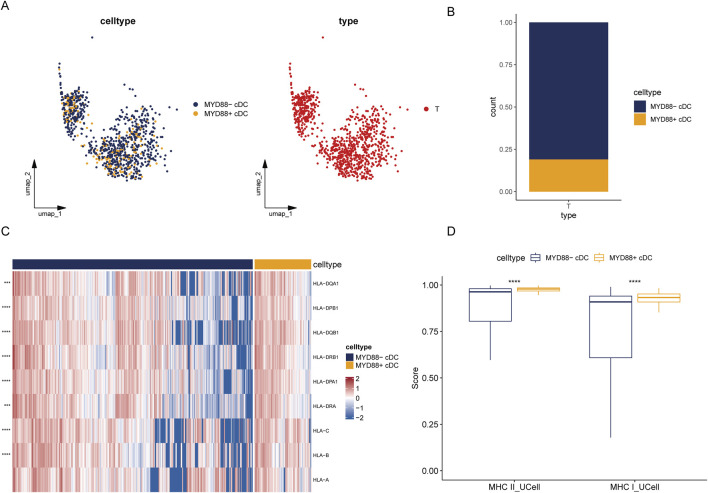
Heterogeneity of tumor cDC. **(A)** UMAP plot of tumor MYD88 cDCs; **(B)** Stacked bar plot of the proportion of tumor MYD88 cDCs; **(C)** Heatmap of MHC class II and MHC class I molecule genes; **(D)** UCell single-cell scoring for MHC class II and MHC class I molecules.

We selected cDCs from tumor samples in the single-cell dataset and categorized cDCs expressing MYD88 at levels greater than 0 as MYD88+ cDCs, while those lacking MYD88 expression were designated as MYD88-cDCs (Figure 5A). In tumor samples, the proportion of MYD88-cDC subpopulations was significantly higher (Figure 5B).

Next, we analyzed antigen-presenting molecule enrichment profiles within different cDC subpopulations and calculated single-cell scores using the UCell package. The MYD88+ DC subpopulation exhibited significantly higher scores for both MHC class I and MHC class II molecules compared to the MYD88- DC subpopulation. Additionally, MYD88+ DCs demonstrated considerably higher expression levels of genes encoding MHC class II molecules, including HLA-DPB1, HLA-DPA1, HLA-DQA1, HLA-DQB1, HLA-DRB1, and HLA-DRA. Furthermore, MYD88+ DCs displayed increased expression levels of genes associated with MHC class I molecules, such as HLA-A, HLA-B, and HLA-C (Figures 5C, D). These findings suggest that MYD88+ DCs may possess enhanced antigen presentation capabilities, enabling them to activate T cells more effectively and play a crucial role in the immune response.

To confirm differential gene expression between the two cell subpopulations, we utilized the FindMarkers function from the Seurat package, applying screening thresholds of p_val_adj <0.05 and |avg_log2FC| > 0.5. Upon comparison, we identified 231 differential genes (231 upregulated; 0 downregulated) between MYD88+ cDCs and MYD88-cDCs. Subsequently, we performed GO and KEGG enrichment analyses on the upregulated differential genes. KEGG analysis revealed enrichment in pathways such as focal adhesion and the PD-L1 expression and PD-1 checkpoint pathway in cancer. Meanwhile, GO Biological Process analysis indicated enrichment for processes including activation of the immune response, T cell receptor signaling pathway, and regulation of antigen receptor-mediated signaling pathways (Supplementary Figure S5).

### 3.6 Cellular communication between cDC cells and T cells in the tumor microenvironment

To further elucidate the mechanisms of cellular communication among different cellular subpopulations, this study employed the R package CellChat (version 1.6.1) to infer intercellular communication networks. The methodology involved constructing a comprehensive database of signaling molecule interactions, considering known structural components of ligand-receptor interactions. These included multimeric ligand-receptor complexes, soluble agonists and antagonists, as well as stimulatory and inhibitory membrane-bound co-receptors.

CellChat employs mass action models, differential expression analyses, and statistical tests on groups of cells to infer cell state-specific signaling communications within scRNA-seq data. Additionally, CellChat provides various visual outputs and quantitative characterizations, facilitating the comparison of intercellular communication through social network analysis tools, pattern recognition methods, and machine learning approaches. It calculates communication probabilities at the signaling pathway level by summarizing the interaction probabilities of all ligand-receptor pairs associated with each pathway.

**FIGURE 6:**
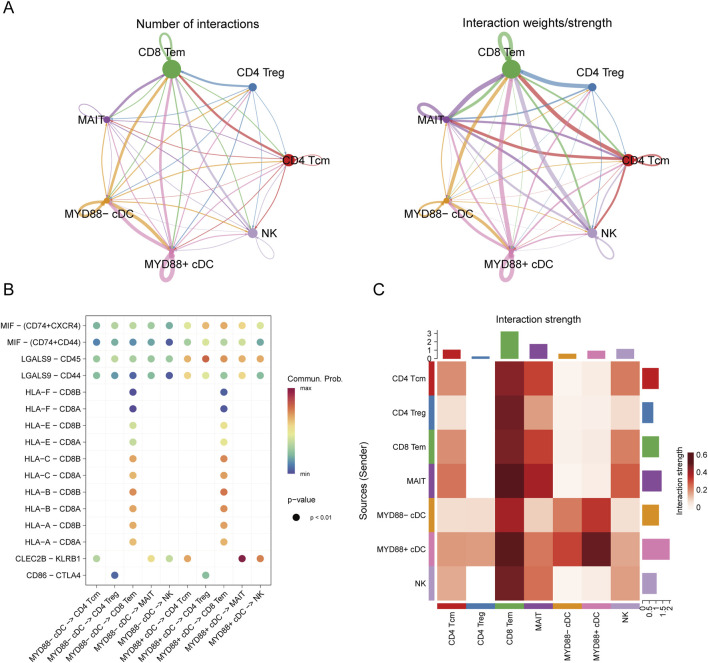
Cell communication analysis. **(A)** Cell communication diagram; **(B)** Bubble plot of cell communication; **(C)** Interaction heatmap of cell communication.

Given the complexity of cellular communication networks, we focused on illustrating the signals transmitted by each subpopulation. To enchance comparability, we adjusted the edge.weight.max parameter, allowing for a clearer comparison of edge weights across different networks. Cellular communication analysis was conducted between cDCs and T cell subpopulations (Figure 6A).

The analysis revealed that MYD88+ cDCs and MYD88-cDCs exhibited distinct interaction strengths with other cell subpopulations across various pathways (Figure 6B). Notably, the results indicated that in ligand-receptor interactions, such as MIF-(CD74+CXCR4), MIF-(CD74^+^CD44), LGALS9-CD45, and LGALS9-CD44, MYD88+ cDCs demonstrated stronger interactions with both CD4^+^ T cells and CD8^+^ T cells (Figure 6C). These findings suggest that MYD88+ cDCs may play a more prominent role in facilitating T cell responses within the tumor microenvironment.

### 3.7 MyD88 promotes H22 tumor growth by reducing T cell function through DCs

**FIGURE 7:**
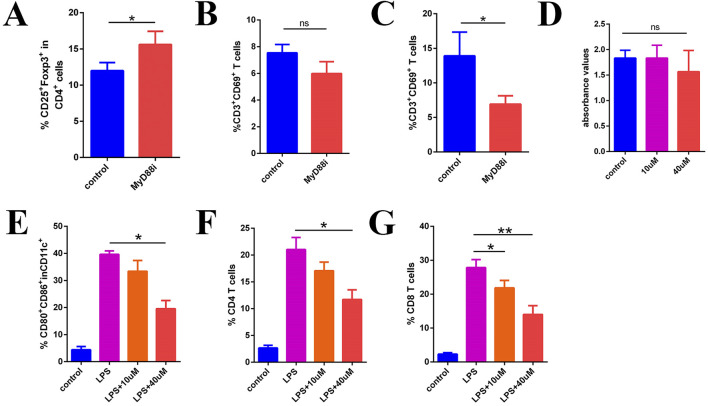
MyD88i inhibits DC maturation and T cell activation. **(A)** The proportion of CD4^+^CD25+Foxp3+ Treg; **(B)** CD3^+^CD69^+^ T cells in the spleen; **(C)** The proportion of CD3^+^CD69^+^ T cells in local lymph node; **(D)** Inhibition of MyD88 in T cells *in vitro* does not appear to have any significant effect on their function; **(E)** The expression of CD80 and CD86 in DCs; **(F, G)** Flow cytometry measured the proliferation of CD4^+^ T cells and CD8^+^ T cells.

To further investigate the impact of MyD88 inhibition on immune cell populations, we examined *in vivo* immune cell markers, with a specific focuse on T cell activation and regulatory T cell (Treg) populations. Flow cytometry analysis following systemic MyD88 inhibition revealed a statistically significant increase in the proportion of splenic Tregs compared to the control group (Figure 7A; Supplementary Figure S6A; 12.00% ± 0.64% vs. 15.60% ± 1.06%, P < 0.05). Although the proportion of splenic CD3^+^CD69^+^ T cells decreased after MyD88 inhibition, this difference was not statistically significant (Figure 7B; Supplementary Figure S6A; 7.54% ± 0.36% vs. 5.98% ± 0.51%, P > 0.05). However, in the lymph nodes, a significant decrease in CD3^+^CD69^+^ T cells was observed following MyD88 inhibition (Figure 7C; Supplementary Figure S6A; 13.9% ± 1.99% vs. 6.91% ± 0.70%, P < 0.05). These findings suggest that MyD88 inhibition not only increases Treg proportions but also modulates T cell activation *in vivo*, particularly affecting activation in the lymph nodes.

To determine whether the inhibition of local T cell activation *in vivo* directly impacts T cell function, we designed an *in vitro* assay to assess the immediate effects of MyD88 inhibiton on T cell activity. T cells were stimulated using functional antibodies (anti-CD3e and anti-CD8e), with the MyD88 inhibitor introduced simultaneously. T cell proliferation and activity were then evaluated using the BrdU assay. The results indicated that MyD88 inhibition did not directly affect T cell activity (Figure 7D, p > 0.05).

MYD88 is a critical signaling molecule in the TLR signaling pathway, playing a pivotal role in DC function and antigen presentation. As shown in Figure 7E, bone marrow-derived DCs stimulated with LPS exhibited high maturation levels, marked by elevated CD80 and CD86 expression. However, MyD88 inhibition (MyD88i) resulted in a concentration-dependent reduction in CD80 and CD86 expression (Figure 7E; Supplementary Figure S6B; 39.63% ± 0.75% in the LPS group vs. 19.53% ± 1.76% in the LPS+40 µM group, P < 0.05), indicating that MyD88 inhibition suppresses DC maturation.

To further investigate the mechanism underlying impaired T cell activation, we conducted mixed culture experiments. Splenocytes from B6 mice were co-cultured with BALB/c bone marrow-derived DCs following MyD88i for flow cytometric analysis. The results demonstrated that CD4^+^ T cell proliferation was significantly reduced in the mixed culture, particularly in the LPS+40 µM group (Figure 7F; Supplementary Figure S6C; 21.03% ± 1.30% in the LPS group vs. 11.70% ± 1.04% in the LPS+40 µM group, P < 0.05). Similarly, CD8^+^ T cell proliferation was also significantly suppressed, exhibiting a dose-dependent inhibitory effect (Figure 7G; Supplementary Figure S6C; LPS group 27.83% ± 1.36% vs. LPS+10 µM group 21.83% ± 1.27%, P < 0.05; LPS group 27.83% ± 1.36% vs. LPS+40 µM group 14.00% ± 1.50%, P < 0.05). These findings suggest that MyD88 inhibition suppresses DC maturation, leading to impaired T cell activation.

### 3.8 MyD88 inhibition can facilitate the growth of H22 tumors

**FIGURE 8:**
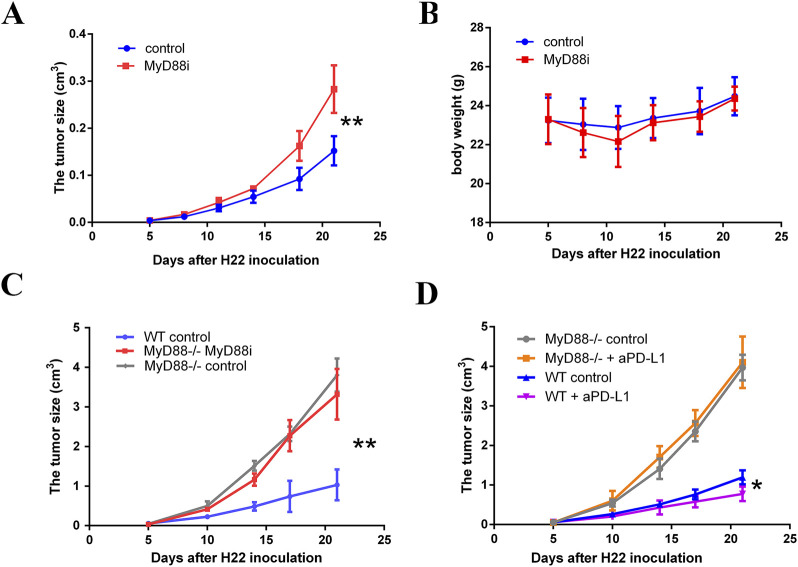
MyD88 inhibition promotes the development of H22 cell-derived tumor. **(A)** The tumor volume and **(B)** body weight were monitored twice per week from day 5 to the end of the observation; **(C)** H22 cell-derived tumors established subcutaneously in MyD88^−/−^ female BALB/c mice (vehicle control, n = 5; 50 mg/kg MyD88i daily from day 5–21, n = 5) or normal female Balb/c mice (vehicle control, n = 5); **(D)** Growth curves are presented comparing MyD88 wild-type female BALB/c mice (vehicle control, n = 3; 200 µg PD-L1 antibody administered every 3 days, n = 3) to MyD88-deficient female BALB/c mice (vehicle control, n = 3; 200 µg PD-L1 antibody administered every 3 days, n = 4).

MyD88 inhibition, achieved through intraperitoneal administration of specific MyD88 inhibitors (Supplementary Figure S6D), resulted in a significant increase in tumor proliferation rates compared to the control group (Figure 8A, P < 0.01). Notably, this increase was not associated with changes in body weight (Figure 8B). In MyD88-deficient female BALB/c mice, H22 tumor growth was significantly accelerated relative to controls (Figure 8C, P < 0.01); however, tumor mass did not differ significantly between the MyD88-deficient group and control groups (Figure 8C, P > 0.05). Importantly, while PD-L1 antibody treatment effectively reduced H22 tumor growth in wild-type mice (Figure 8D, P < 0.05), this therapeutic effect was absent in MyD88-deficient mice (Figure 8D, P > 0.05). These findings suggest that MyD88 plays a pivotal role in mediating the immune response to tumor growth and in determining the efficacy of PD-L1 blockade therapy.

## 4 Discussion

The tumor microenvironment plays a crucial role in shaping the prognosis of HCC. This environment consists not only tumor cells but also various interacting immune cell populations, whose interplay can significantly infulence tumor prgression. Database analyses indicate low MyD88 expression in tumors, consistent with some studies. However, other investigations-including our previous work with the H22 mouse HCC cell line (Liu et al., 2019)-have reported elevated MyD88 expression. This discrepancy highlights the complexity and heterogeneity of HCC, suggesting that MyD88 expression may vary depending on tumor context and microenvironmental factors.

High MyD88 expression in tumors has been significantly associated with prognostic outcomes, including overall survival (OS), as indicated in prior analyses. Our previous work demonstrated that HCC cells with elevated MyD88 expression are susceptible to MyD88 inhibition, which disrupts the tumor cell cycle and subsequently slows tumor growth (Jia et al., 2014). Furthermore, MyD88 plays a critical role in tumor proliferation and metastasis, primarily through the PI3K/Akt signaling pathway and the epithelial-mesenchymal transition (EMT) process (Jia et al., 2014). Other studies have also reported that highly malignant cells exhibit elevated MyD88 levels, suggesting that increased MyD88 expression may drive hepatocellular carcinoma progression (Ochi et al., 2012b). Furthermore, MyD88 knockdown has been shown to enhance the sensitivity of intestinal epithelial cells to azoxymethane (AOM)/dextran sodium sulfate (DSS) treatment by inhibiting IL-18 receptor-mediated signaling, a pathway implicated in the development of colitis-associated colon cancer (Salcedo et al., 2010). These findings have important clinical implications, highlighting MyD88 as a potential therapeutic target. However, while these studies underscore MyD88’s significant role in tumor biology, the precise mechanistic underpinnings remain incompletely understood. We further investigated the role of MyD88 in modulating immune function within the tumor microenvironment, a potential key mechanism underlying tumor progression and therapeutic response. Immune responses play a critical role in shaping the tumor microenvironment (Baharom et al., 2022). Our analysis identified differential MyD88 expression across various immune cell populations, including NK cells, Th2 cells, memory CD8^+^ T cells, and memory CD4^+^ T cells. Notably, activated CD8^+^ T cells exhibited a negative correlation with MyD88 expression, whereas memory CD4^+^ and memory CD8^+^ T cells showed positive correlations, suggesting a complex interplay between MyD88 signaling and adaptive immune responses. MyD88 may exert diverse effects on immune cells within the tumor microenvironment, potentially influencing patient outcomes. However, its expression does not uniformly impact all immune cell types. Correlation analyses revealed a significant association between MyD88 expression and the functionality of cDCs (Macri et al., 2018), with MyD88+ cDCs exhibiting enhanced antigen presentation and T cell activation capabilities. This enhancement contribute to antitumor immunity and influence clinical outcomes in HCC. Conversely, MyD88 inhibition disrupts dendritic cell function, leading to increased Th2 differentiation, inflammation, and carcinogenesis (Ochi et al., 2012a). In contrast, uninhibited MyD88 supports normal dendritic cell function, fostering effective immune responses and tumor control.

DCs, among the most potent antigen-presenting cells, are influenced by various factors that regultate their functionality (Jhunjhunwala et al., 2021). High expression of MHC class I and MHC class II molecules enhances their antigen-presenting capacity, a phenomenon associated with MyD88 expression (Das et al., 2014). The TLR/MyD88 signaling pathway plays a critical role in DC maturation and function. Studies have shown that LPS-induced activation of this pathway promotes DC maturation, increasing the expression of costimulatory molecules such as CD80 and CD86. However, MyD88 blockade inhibits this maturation and functional enhancement. Our analysis further revealed that MyD88+ cDCs exhibit stronger interactions with both CD4^+^ T cells and CD8^+^ T cells, suggesting that MyD88 may serve as a biomarker of DC activation, facilitating anti-tumor immunity.

In summary, our analysis indicates that MYD88 is associated with the clinical prognosis of HCC and may influence clinical outcomes through cDCs. Both *in vivo* and *in vitro* experiments have demonstrated that MYD88 activates T cells via DCs, thereby promoting anti-tumor immunity. Notably, immune checkpoint inhibitors can enhance the anti-tumor effects of MYD88 or act synergistically to improve therapeutic efficacy. However, the precise mechanisms by which MYD88+ cDCs influence tumor progression within the tumor microenvironment warrant further investigation.

## Data Availability

The original contributions presented in the study are included in the article/Supplementary Material, further inquiries can be directed to the corresponding authors.
