# Supplementary figures and images for: Dendritic cell-derived MYD88 potentiates as a biomarker for immune regulation in hepatocellular carcinoma and may predict a better immunological result
Source: Front Cell Dev Biol. 2025 Mar 24;13:1554705. doi: 10.3389/fcell.2025.1554705 (PMC11973264; doi:10.3389/fcell.2025.1554705)

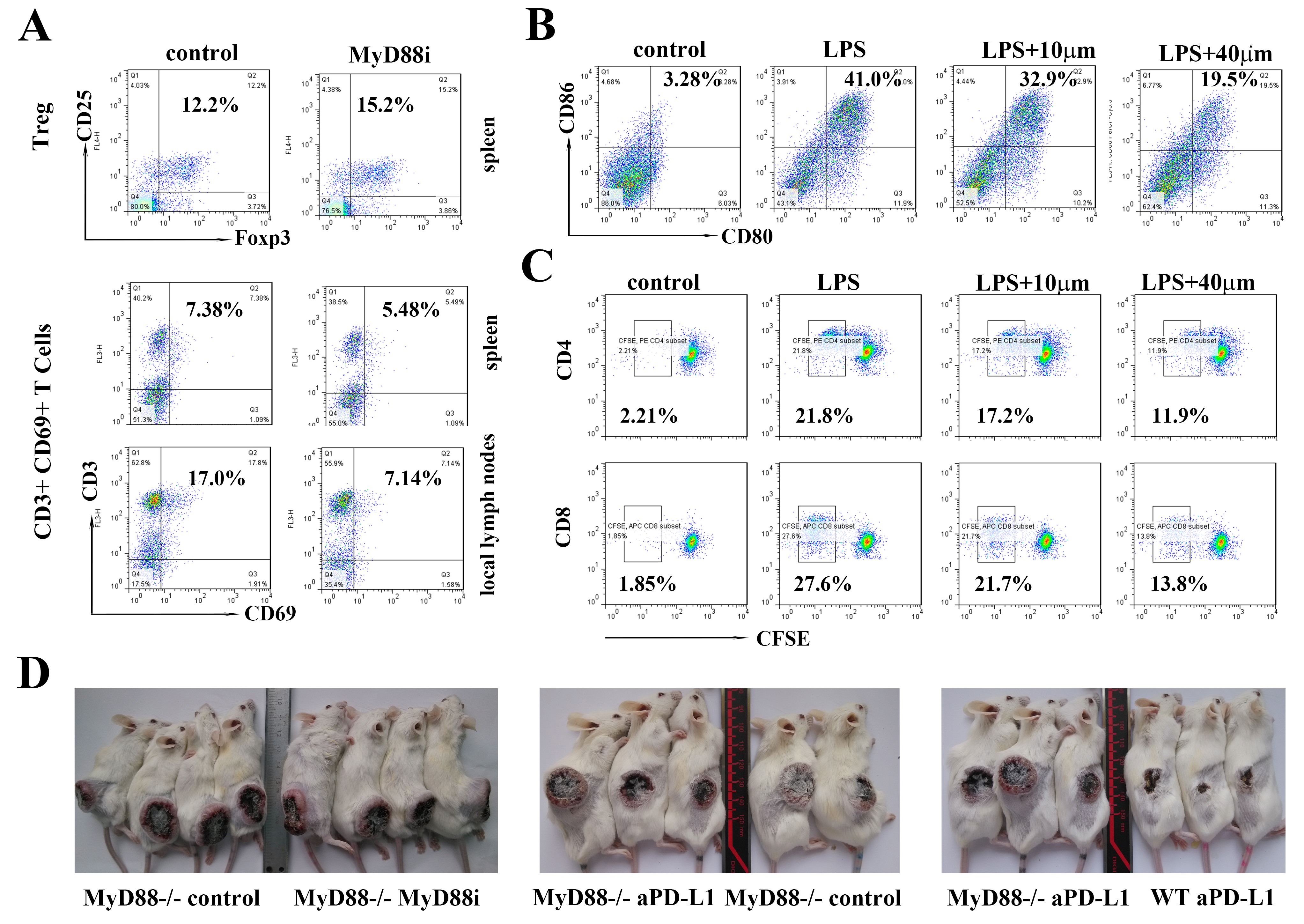

Supplement: Supplementary file 1 [file Image6.tif]

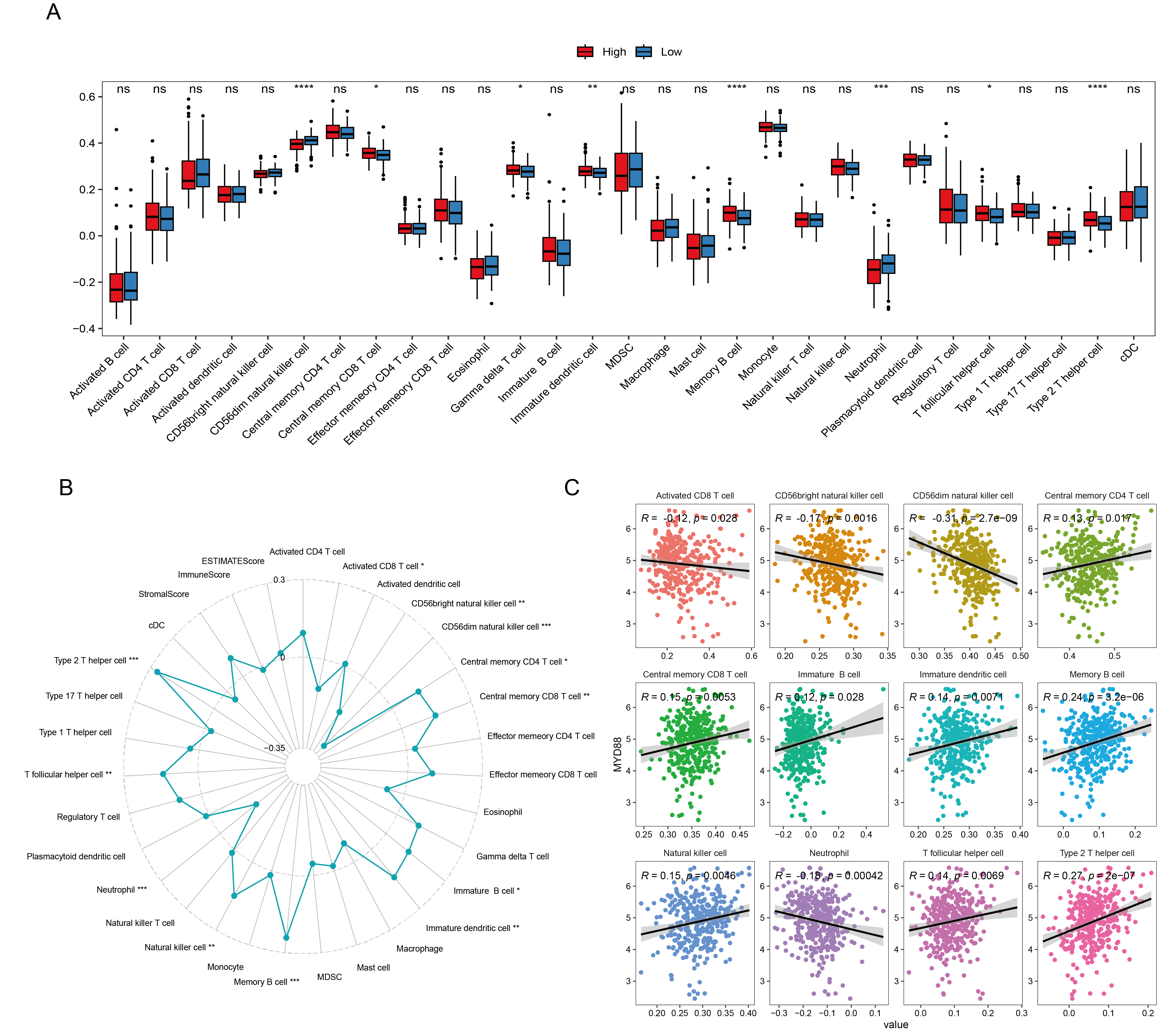

Supplement: Supplementary file 2 [file Image3.tif]

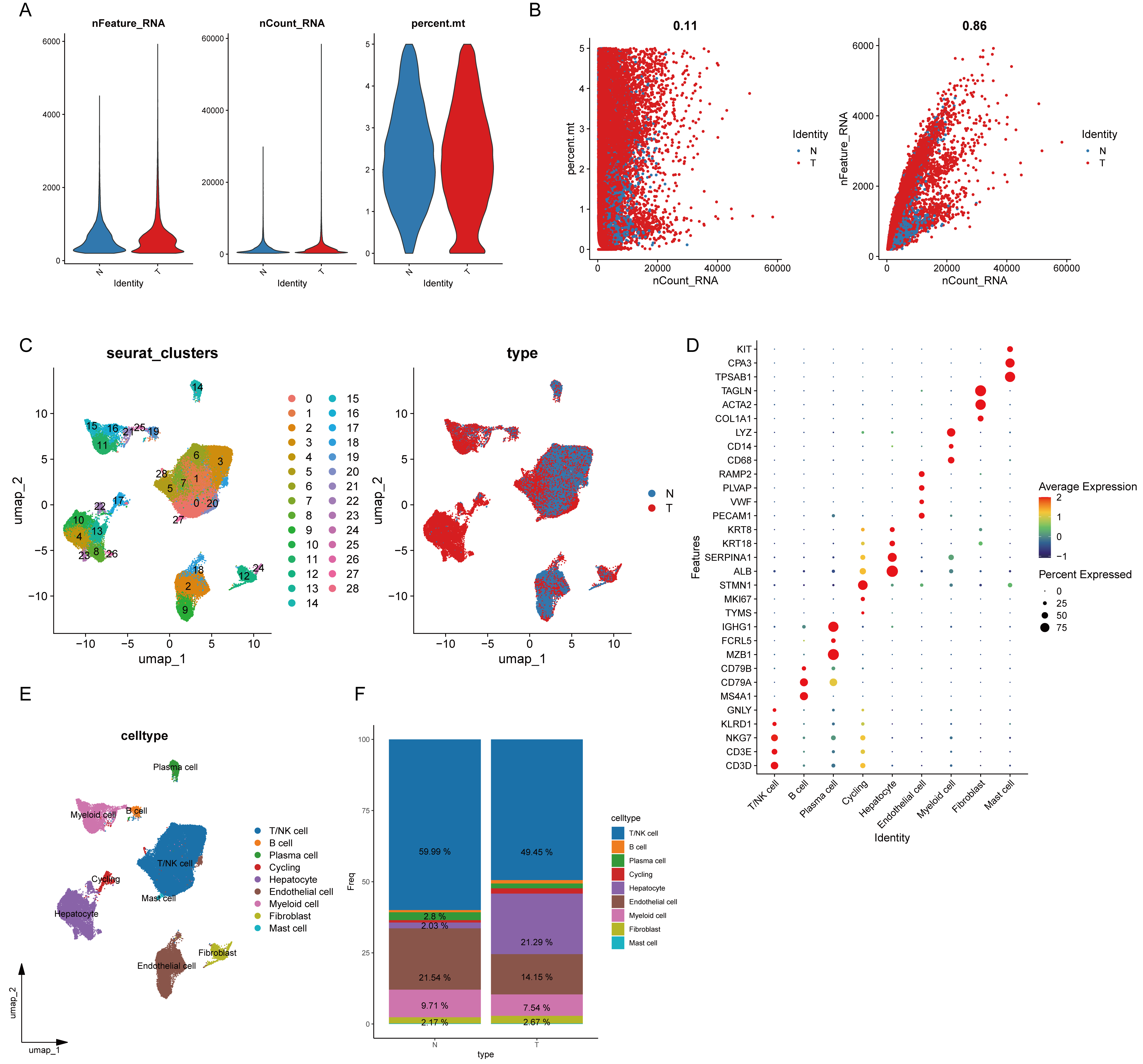

Supplement: Supplementary file 3 [file Image4.tif]

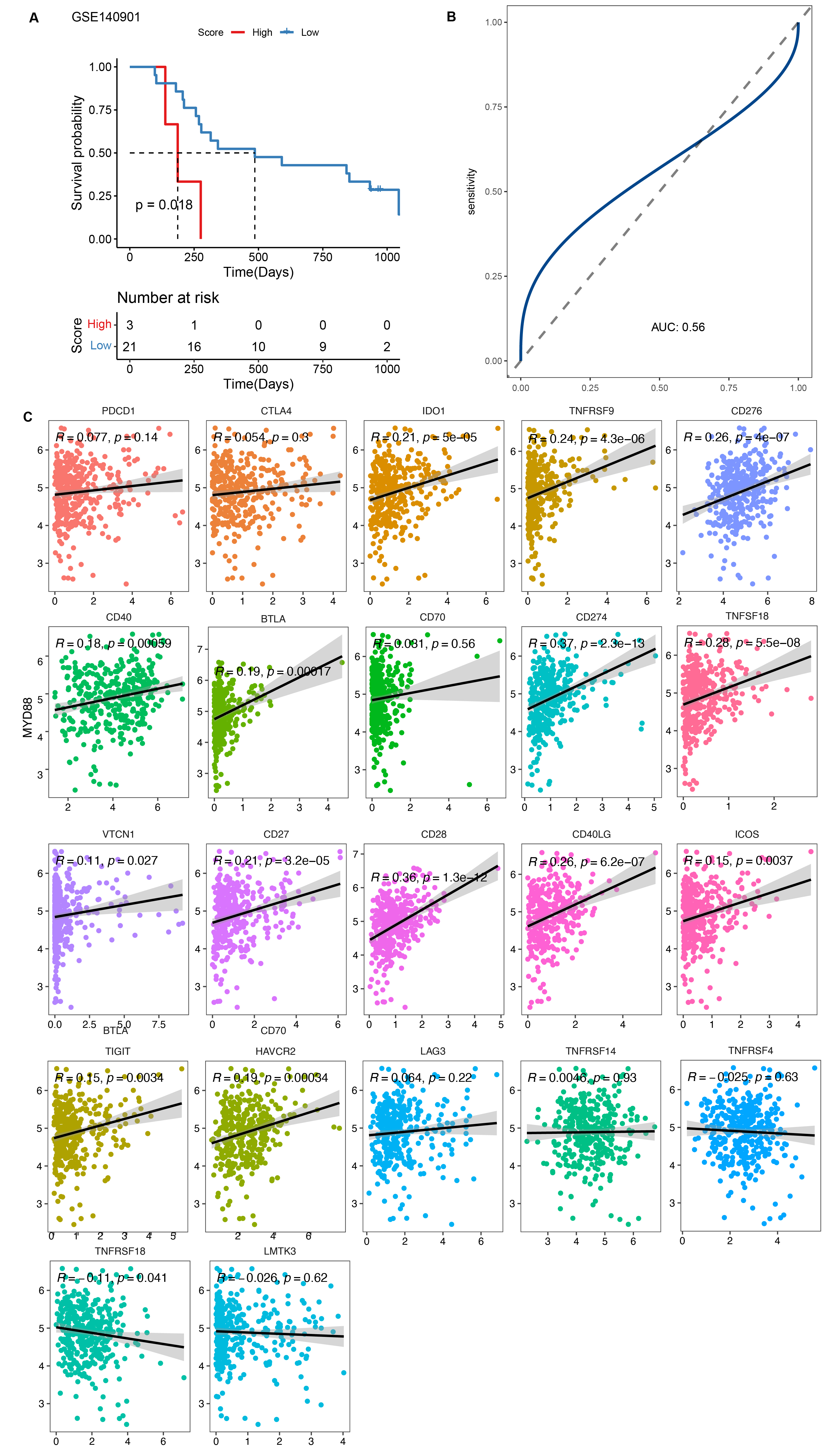

Supplement: Supplementary file 4 [file Image2.jpeg]

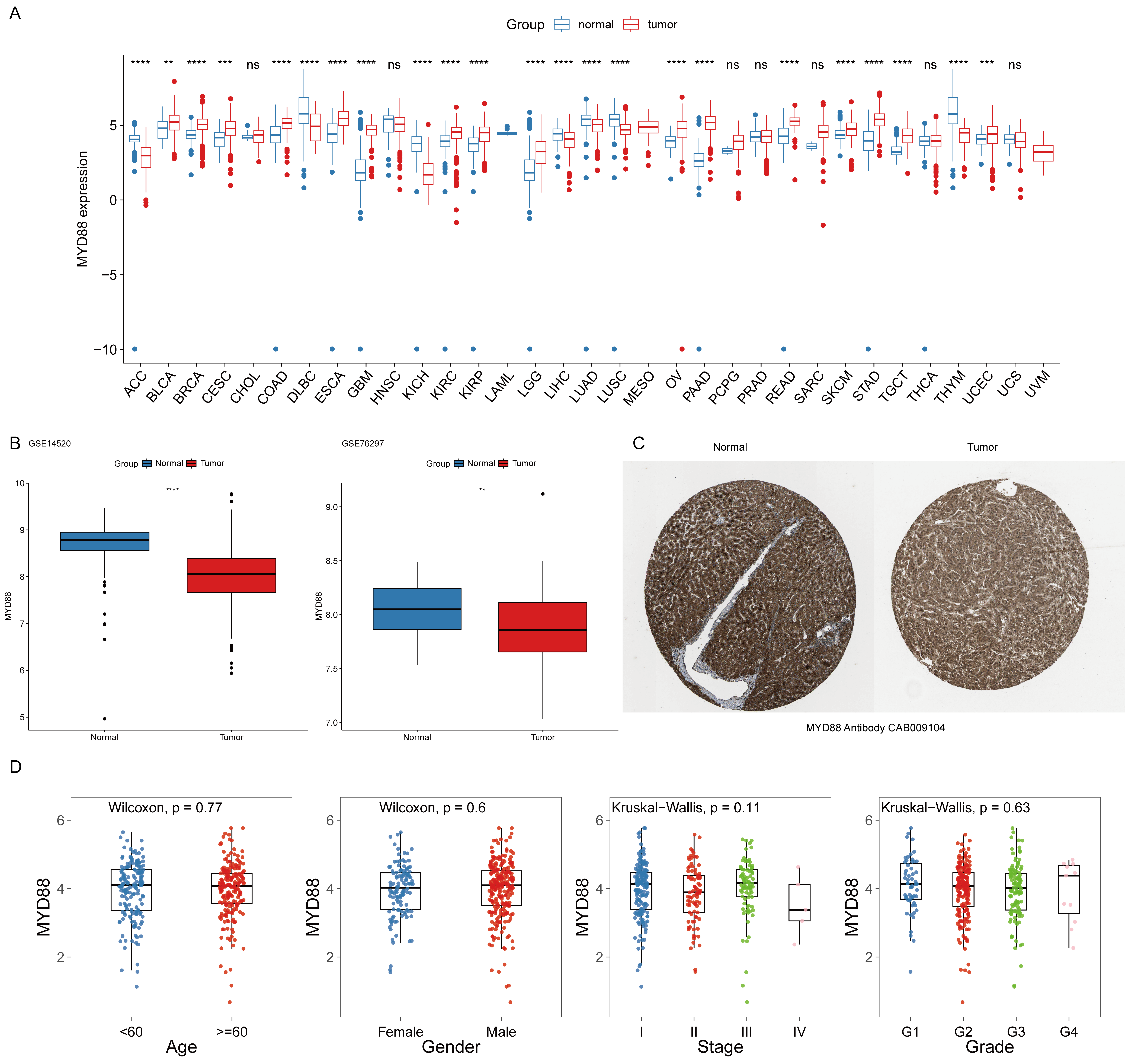

Supplement: Supplementary file 5 [file Image1.tif]

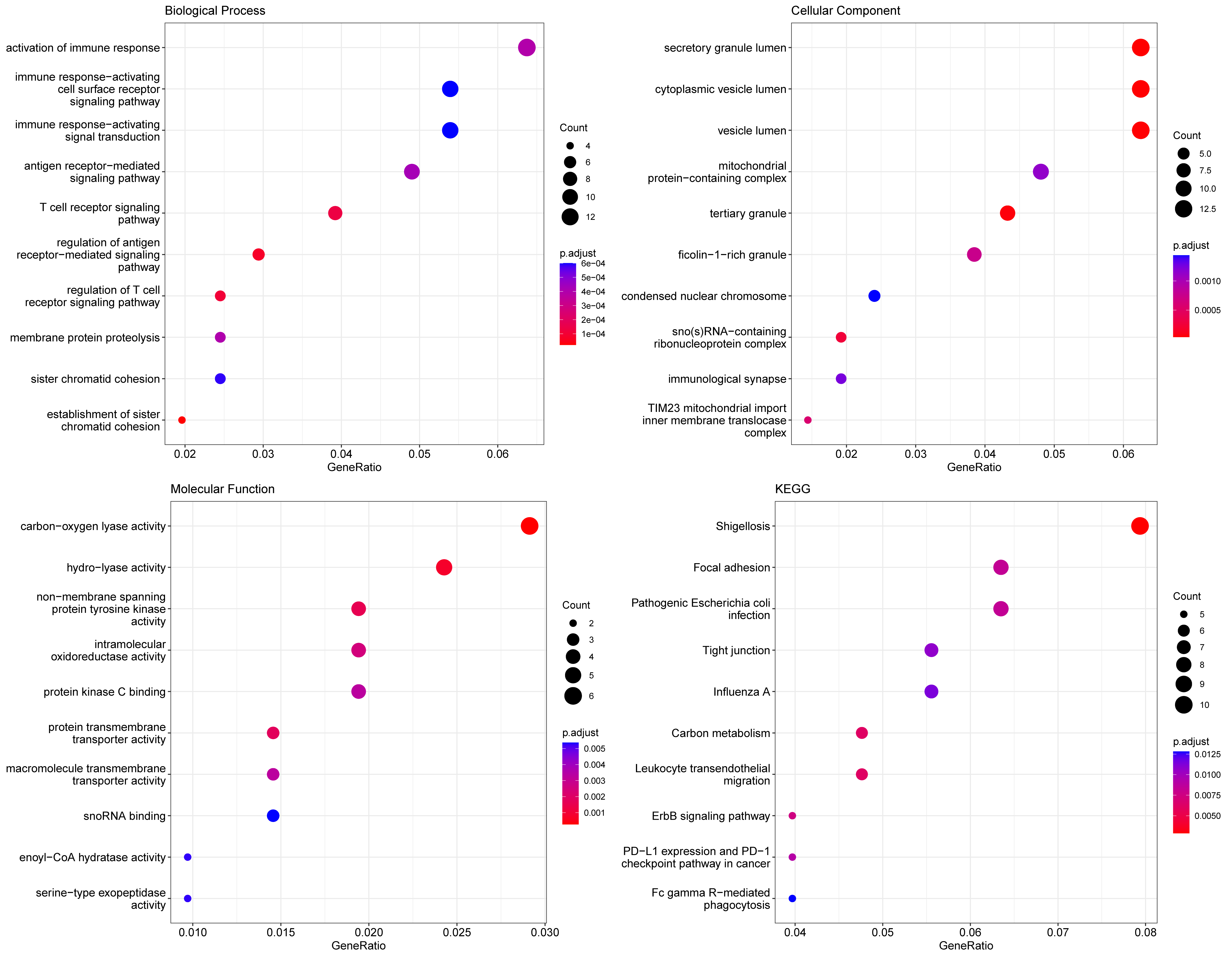

Supplement: Supplementary file 6 [file Image5.tif]
