# Supplementary material for: Dendritic cell-derived MYD88 potentiates as a biomarker for immune regulation in hepatocellular carcinoma and may predict a better immunological result
Source: Front Cell Dev Biol. 2025 Mar 24;13:1554705. doi: 10.3389/fcell.2025.1554705 (PMC11973264; doi:10.3389/fcell.2025.1554705)
Supplement: Supplementary file 7 [file DataSheet1.docx]

**Relationship Between MYD88 Expression and Immunotherapy Efficacy in HCC**

We collected data from 13 hepatocellular carcinoma (HCC) patients who received neoadjuvant immune checkpoint inhibitors (ICIs**,** PD-1 inhibitors) therapy at Renmin Hospital of Wuhan University between February 2023 and February 2025 (dosage regimen: 200mg/q3w). MYD88 expression was analyzed in tumor pathological tissue samples from these patients. Patient grouping was based on the proportion of residual tumor cells, as determined by the pathological report assessing neoadjuvant efficacy. Following the MP classification criteria for neoadjuvant response, a 90% reduction in tumor cells was used as the threshold for a significant therapeutic effect. Accordingly, patients were categorized using a 10% residual tumor cell threshold: the immune response group (IRG; residual tumor cells ≤10%, n=7) and the low immune response group (LIRG; residual tumor cells >10%, n=6). Immunohistochemical analysis was performed using a NanoZoomer® S360 (HAMAMATSU PHOTONICS) white light scanner, and images were analyzed with ImageJ software at a magnification of 40×10.


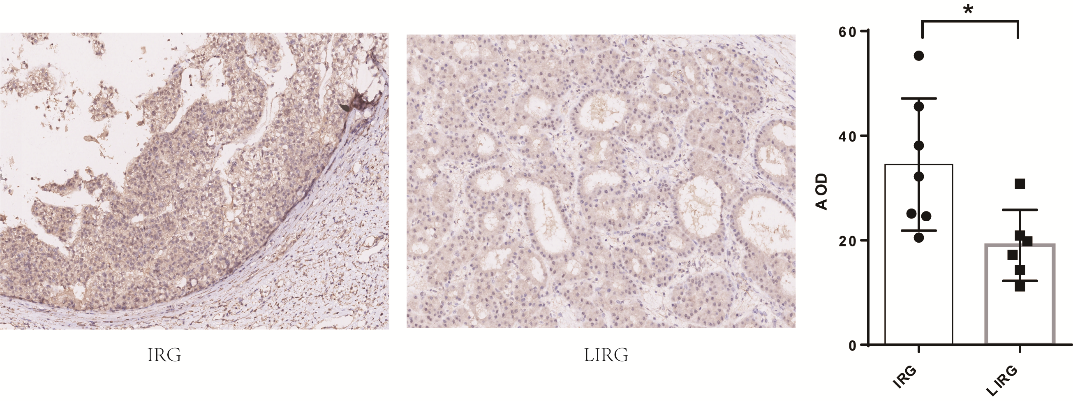


Immunohistochemical staining and Average Optical Density (AOD, area%).

AOD=IOD/area, IOD is Integrated option density for short.
